# The essential elements of Alzheimer’s disease

**DOI:** 10.1074/jbc.REV120.008207

**Published:** 2020-11-27

**Authors:** Peng Lei, Scott Ayton, Ashley I. Bush

**Affiliations:** 1 Department of Neurology and State Key Laboratory of Biotherapy, West China Hospital, Sichuan University, and Collaborative Innovation Center for Biotherapy, Chengdu, P.R. China; 2 Melbourne Dementia Research Centre, Florey Institute of Neuroscience and Mental Health, The University of Melbourne, Victoria, Australia

**Keywords:** zinc, copper, iron, selenium, ferroptosis, Alzheimer’s disease, clioquinol, PBT2, Aβ, amyloid-β peptide, AD, Alzheimer’s disease, ApoE, apolipoprotein E, APP, amyloid protein precursor, CSF, cerebrospinal fluid, Cp, ceruloplasmin, DFO, deferoxamine, FAD, familial AD, GPx4, glutathione peroxidase 4, HO-1, heme oxygenase 1, LTP, long-term potentiation, MRI, magnetic resonance imaging, NAC, N-acetylcysteine, NMDA, N-methyl-D-aspartate, PBT2, 5,7-dichloro-2-[(dimethylamino)methyl]quinolin-8-ol, PUFA, polyunsaturated fatty acids, QSM, quantitative susceptibility mapping, RTA, radical trapping agent, SOD1, superoxide dismutase 1, ZIP, Zinc regulated transporter-like Iron regulated transporter-like Protein, ZnT, Zinc transporter

## Abstract

Treatments for Alzheimer’s disease (AD) directed against the prominent amyloid plaque neuropathology are yet to be proved effective despite many phase 3 clinical trials. There are several other neurochemical abnormalities that occur in the AD brain that warrant renewed emphasis as potential therapeutic targets for this disease. Among those are the elementomic signatures of iron, copper, zinc, and selenium. Here, we review these essential elements of AD for their broad potential to contribute to Alzheimer’s pathophysiology, and we also highlight more recent attempts to translate these findings into therapeutics. A reinspection of large bodies of discovery in the AD field, such as this, may inspire new thinking about pathogenesis and therapeutic targets.

Alzheimer’s disease (AD), the most common form of dementia, is increasingly prevalent and a worsening healthcare burden. The cognitive deterioration of AD has remained frustratingly recalcitrant to candidate disease-modifying therapeutics despite massive efforts over the last 35 years. Most research into therapeutics has been philosophically guided by the connection of the hallmark histopathology of AD, cortical amyloid plaques, and neurofibrillary tangles, with familial dementia-causing mutations associated with their most insoluble component proteins, the amyloid-β peptide (Aβ) (1, 2), and the microtubule-associated protein tau (3, 4). Both proteins are normal and soluble components of tissue that become denatured by events that are not simply related to overproduction.

Alois Alzheimer himself came to the conclusion 5 years after his description of plaques and tangles that despite their dramatic appearance, these histopathologies were not the cause of neurodegeneration in AD but, rather, a signature epiphenomenon (reviewed [5]). Yet, dogged efforts have been made in the modern era to causatively link the aggregation of these proteins to the brain atrophy, synaptic disintegration, and neuronal loss that characterize AD, through death mechanisms that remain unproven after decades of research (*e.g.,* the Amyloid Cascade Hypothesis [6]). The discovery of familial AD (FAD) causative mutations of the amyloid protein precursor (APP) and the presenilins (that cleave the carboxyl terminus of Aβ from APP) as well as mutations of tau that cause fronto-temporal dementia have been interpreted simplistically through the prism of the toxic proteinopathy theory. Efforts to investigate the neurodegeneration mechanisms of the genetic lesions of AD outside of the formation of putatively toxic aggregates have received, in our opinion, insufficient attention, for example, that pathogenic presenilin mutations cause neurodegeneration without proteinopathy through a loss of trophic function (7). Indeed, the dogma that all FAD causative mutations of the presenilins generate longer proaggregate forms or more Aβ has been persuasively challenged as data to the contrary accumulate (5, 8).

Billions of dollars are being spent by big pharmaceutical companies on lowering Aβ or tau as therapeutic strategies. This approach was justified on the premise of the earliest data from murine knockouts of APP and tau, which have minimal phenotypes in youth, leading to the conclusion that these proteins therefore must be functionless rogues that humans can live without. But, the safe redundancy of tau and APP is unlikely because the adverse phenotypes relevant for neurodegeneration, particularly those related to brain metal dyshomeostasis, are, like AD itself, a product of aging and do not emerge until the postreproductive epoch (9, 10, 11, 12). Nonetheless, clinical trials of Aβ-lowering agents proceed despite more than 30 phase 3 trials failing to demonstrate conspicuous cognitive benefit to AD patients or sometimes being harmful, even upon successful clearance of amyloid plaques (13, 14, 15, 16, 17, 18). One of these, aducanumab, has been presented for registration to the Food and Drug Administration on the basis of debatable benefits that were seen in one but not both of its two phase 3 trials and could be explained as a placebo effect caused by the unblinding when treatment is temporarily suspended upon activation of the amyloid related imaging artefact protocols, which is much more common in the active arm (19). In no instance has an amyloid-lowering treatment shown a reliable and indisputable benefit.

With amyloid being challenged as a therapeutic target, other neurochemical changes in AD have attracted growing interest. Hence, the subject of this monograph. Biometals such as zinc, copper, and iron, which have essential roles in normal physiology, have been implicated in AD pathogenesis for more than 25 years, while commanding a tiny fraction of the research and clinical trial resources committed to proteinopathy research. These are physiologically essential metal ions, but their nutritional (or genetic) dysregulation causes neurotoxicity and neurological damage. These metal ions are stringently regulated by multiple handling systems because excess can also be neurotoxic. These should not be called “trace” metal ions because their concentrations in the brain are within the same order of magnitude as magnesium. Also, the epithet “heavy metal” should not be applied to these versatile and essential metal ions but should be reserved for metals such as lead, mercury, and cadmium that are conspicuously neurotoxic and serve no biochemical purpose. Although aluminum has been investigated as a neurotoxicant that may influence AD, we place it outside of this review of essential elements because it is a nonessential “light” metal with no biochemical function but is very abundant in the environment (present at low micromolar concentrations in plasma as an environmental contaminant) and only potentially toxic at high concentrations (20).

Ionic zinc was first reported in 1994 to induce histological amyloid structures rapidly out of soluble Aβ (21). Later, ionic copper and iron were found to facilitate Aβ aggregation as well as catalyze reactive oxygen species generation from the ternary complexes (22, 23, 24, 25, 26). Over this time, evidence has accumulated to indicate that these biological elements impact Aβ and tau production, posttranslational modification, aggregation, and toxicity. Sensitive multielement assay technology, *e.g.,* inductively coupled plasma mass spectrometry, has enabled metallomics (“elementomics”, actually, because the array of elements measured simultaneously frequently includes nonmetals, such as selenium [Se]) to be adapted to examining biological samples. Furthermore, biological metal dyshomeostasis alone has been shown to cause neuronal and cognitive dysfunction. Here, we review the associations of the brain’s three most abundant physiological transition metals, iron, zinc, and copper, with the pathophysiology and neuropathology of AD. Because the iron-dependent regulated cell death pathway, ferroptosis, is so closely involved with the selenoenzyme glutathione peroxidase 4 (GPx4) (27, 28), we also discuss the role of the essential trace metal Se.

## Zinc

Zinc is essential for brain function, and it participates in protein structure stabilizing and catalytic reactions in living organisms. It is concentrated in the gray matter of the brain, where 20 to 30% of brain zinc is located in glutamatergic vesicles (29), which results in an extraordinary level of Zn^2+^ in the synaptic cleft during neurotransmission (100–300 μM) (30, 31). The high flux of zinc in the synapse contributes to synaptic plasticity, and long-term potentiation (LTP) in the hippocampal CA3 region is modulated by zinc at presynaptic and postsynaptic targets (32). Synaptic zinc turnover is therefore highly energetic but fatigues with age (33), highlighting the potential for zinc dysregulation to contribute to cognitive impairment in AD. Zinc homeostasis is mostly regulated by the SLC39 family (zinc regulated transporter-like iron regulated transporter-like proteins, ZIPs), which has 14 members that transport Zn^2+^ into the cytoplasm (from organelles and cellular uptake), and the SLC30 family (zinc transporters, ZnTs), which has 10 members that transport Zn^2+^ out of the cytoplasm (extracellularly and into organelles) (34). These two families of transporters are believed to be relative selective for Zn^2+^, but a few ZIPs and ZnTs transport other metals, such and Fe, Mn, and Cd. The expression of various members of these families is tissue-specific. ZnT3 expression is selectively expressed in cortical tissue, accounting for 20% of total brain zinc content, and, by loading Zn^2+^ into glutamatergic synaptic vesicles, is responsible for the high concentrations of extracellular Zn^2+^ released during neurotransmission (35). ZnT3 and its associated synaptic Zn^2+^ release is strongly implicated in deteriorating cognitive function in AD and in the pathogenesis of the hallmark amyloid pathology.

**Figure 1:**
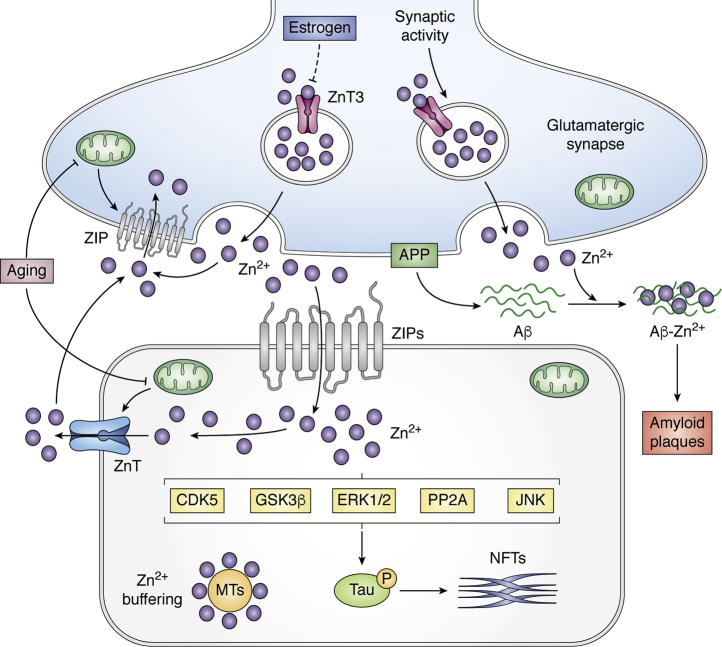
**Neuronal zinc homeostasis is dysregulated in Alzheimer's disease.** Zn^2+^ enters neuronal cytoplasm through ZIPs, whereas efflux from the cytoplasm is regulated by ZnTs. There are many types of ZIPs and ZnTs expressed in neurons, but ZnT3 is implicated in cognitive loss with aging and amyloid formation in AD. ZnT3 concentrates Zn^2+^ in glutamatergic synaptic vesicles that is released upon synaptic activity and then is normally rapidly taken up by unidentified energy-dependent mechanisms. During aging, mitochondrial energy is decreased, leading to more sluggish reuptake of extracellular Zn^2+^. Loss of estrogen, as occurs during menopause, increases ZnT3 protein levels, potentially increasing Zn^2+^ release. Extracellular Zn^2+^ binds to Aβ and induces its aggregation, becoming trapped in the amyloid. Intracellularly, metallothioneins, as the major Zn^2+^-buffering peptides, maintain free Zn^2+^ at appropriate levels, but neuronal Metallothionein III levels are depleted in AD. Increased cytoplasmic free Zn^2+^ enhances tau phosphorylation by activating CDK5, GSK3β, ERK1/2, or JNK kinases and by inhibiting PP2A activity. Aβ, amyloid β; APP, amyloid precursor protein; CDK5, cyclin-dependent kinase 5; ERK1/2, extracellular signal-regulated protein kinase 1/2; GSK3β, glycogen synthase kinase 3β; JNK, c-Jun N-terminal kinase; MTs, metallothioneins; NFTs, neurofibrillary tangles; PP2A, protein phosphatase 2A; ZIPs, zinc regulated transporter-like iron regulated transporter-like proteins; ZnTs, zinc transporter proteins.

After the discovery that Aβ is normally secreted by neurons as a soluble peptide (36), factors inducing Aβ aggregation became of interest. Zn^2+^ was found to bind to Aβ with affinity in the high nM to low μM range and to induce its rapid aggregation and precipitation (21, 37), with up to 3 eq. of Zn^2+^ per mole of Aβ in the precipitate (38). Histidine–Zn^2+^ bridges mediate the reversible assembly of these precipitates (21, 37), and Asp7 is also essential for the interaction (39), which can be abolished by phosphorylation (40). The metal binding site on Aβ is not specific for Zn^2+^ and overlaps with residues that coordinate (and reduce) Cu^2+^ and Fe^2/3+^ (*vide infra*). The complex of Aβ–zinc is resistant to proteolysis (37), promoting the stability of Aβ aggregates (Fig. 1). Importantly, the rat/mouse homolog of Aβ is exceptional among mammalian sequences for having a His13Arg substitution that attenuates Zn^2+^ binding and Zn^2+^-induced precipitation (21), which may help explain why these rodents do not develop amyloid plaques (41) unless made transgenic to overexpress the human Aβ sequence. These substitutions also impair the binding of Cu^2+^ and Fe^3+^ at an overlapping binding site (*vide infra*). Curiously, APP possesses an ectodomain high-affinity zinc-binding site remote from the Aβ sequence that promotes the affinity of APP for heparin (42, 43). Little is known of the physiological purpose of this site, although it overlaps with a palmitoylation site that modulates APP binding and hence cleavage to generate the N-terminus of Aβ (44, 45).

Zn^2+^ can induce different forms of Aβ aggregates depending on the ratio between Aβ and zinc: stoichiometric concentrations of zinc induce nonfibrillary aggregates enriched in the reversible α-helical structure, whereas fibrillar, β-sheet–enriched aggregates are formed with substoichiometric concentrations of zinc as a consequence of seeding (46, 47). This is a major differentiation between the fibrillar pathway of amyloid formation that occurs with a micromolar concentration of peptide *in vitro* at a slow rate through hydrophobic β-sheet forces, taking days, compared with the millisecond reversible precipitation of Aβ by Zn^2+^, mediated by an ionic histidine bridge (21, 22, 48, 49, 50, 51, 52, 53, 54). Zn^2+^ can compete with Cu^2+^ for Aβ, silencing its redox activity and peroxide formation and suppressing oxidation in the vicinity of plaques (55).

As evidence for Zn^2+^ aggregating soluble Aβ *in vivo*, zinc accumulates in plaques in AD and may be as high as 1 mM in this vicinity (26, 56, 57). This is also seen in animal models of AD, where zinc is elevated in plaques of *APP*/*PS1* mice determined by Timm’s stain (58) as well as X-ray fluorescence microscopy (59), plaques of Tg2576 mice determined by metallomic imaging mass spectrometry (60), and plaques within the amygdala of aged (over 23-years-old) macaques (61). Furthermore, chelators dissolve insoluble Aβ deposits while releasing Zn^2+^ from postmortem AD-affected brain tissue samples (24, 62). Further evidence for extracellular Zn^2+^ inducing amyloid formation comes from the effects of *ZnT3* knockout in suppressing interstitial and vascular amyloid pathology in APP transgenic mice (63, 64).

The significance of amyloid plaques themselves in the etiopathogenesis of AD is uncertain. It is understood from both postmortem and PET ligand studies that amyloid deposition commences 1 to 2 decades before the onset of functional impairments in the natural history of AD. However, 30 to 40% of people in the age of risk for AD have conspicuous amyloid pathology without cognitive impairment. Indeed, there is no association of amyloid burden with the rate of premortem cognitive decline (65). With the failure of more than 30 phase 3 clinical trials that lower brain Aβ, it is possible that amyloid plaque pathology might be a biomarker of another process, such as zinc dyshomeostasis. Recent evidence has brought to light a mechanism that may explain amyloid deposition caused by the slow turnover of synaptic Zn^2+^ released during glutamatergic synaptic transmission (Fig. 1). This pool of Zn^2+^ is normally rapidly cleared by regional mechanisms that are still uncertain. Extracellular Zn^2+^ clearance from stimulated rat hippocampal slices is impaired by the advanced age of the donor and female sex, two prominent risk factors for extracellular amyloid pathology, which increase the average extracellular Zn^2+^ concentration over time and promote the aggregation of Aβ (33). In mice, a drop in estrogen (induced by oophorectomy, recapitulating changes in human menopause) increases the levels of ZnT3 protein (66).

Clioquinol (5-chloro-7-iodoquinolin-8-ol) was originally identified as a copper/zinc chelator and ameliorated both amyloid pathology and cognitive loss in APP transgenic models of AD (67, 68). A 36-weeks phase 2 clinical trial of clioquinol for AD significantly slowed deterioration (69), but the drug was supplanted for development by PBT2 (5,7-dichloro-2-[(dimethylamino)methyl]quinolin-8-ol), which was more easily synthesized. Like clioquinol, PBT2 rescued the amyloid burden, lowered phosphorylated tau, and rapidly improved memory performance in the *APP*/*PS1* transgenic model of AD (62).

**Figure 2:**
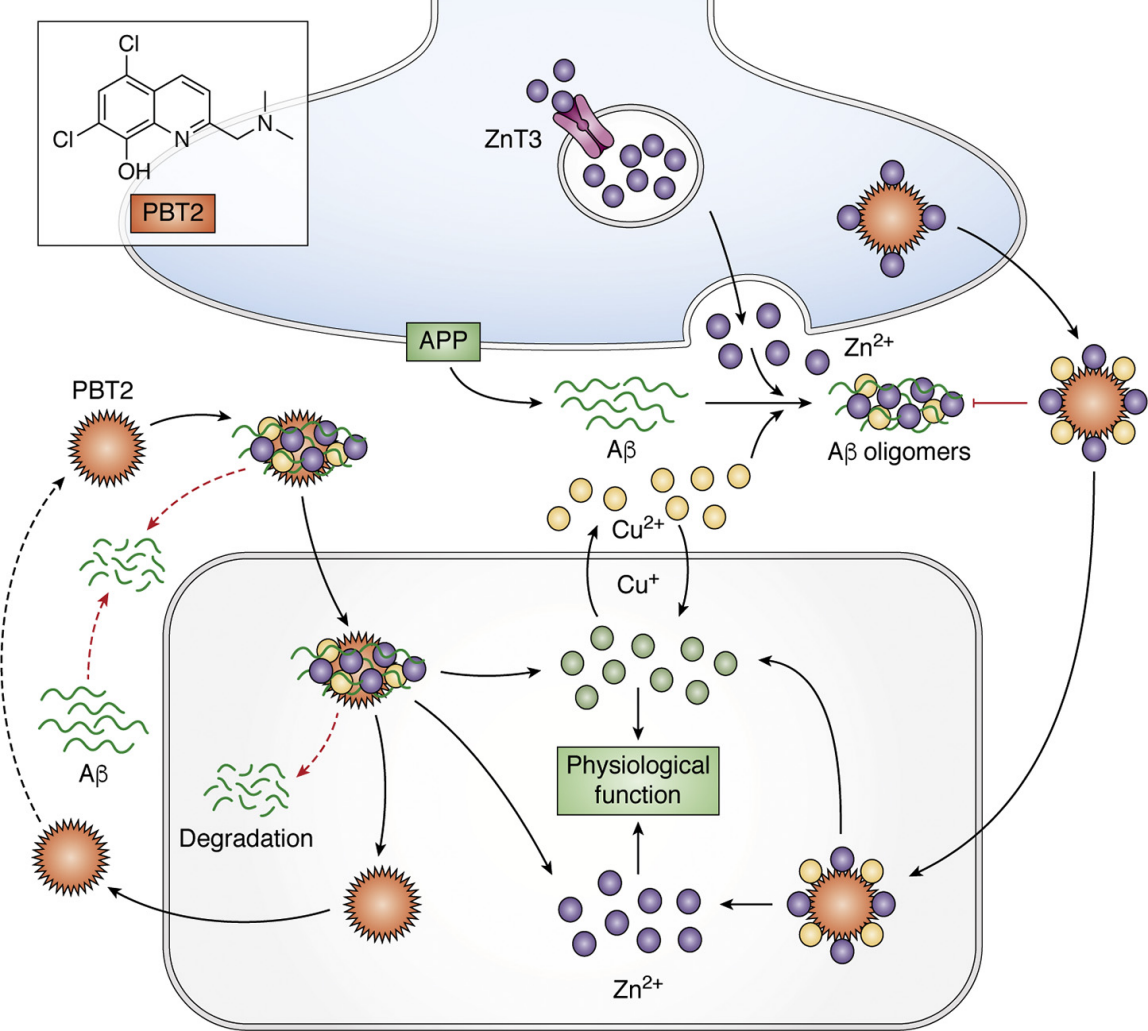
**Potential mechanisms of PBT2 in Alzheimer's disease.** Soluble interstitial Aβ reacts with extracellular Zn^2+^ and Cu^2+^ to form protease-resistant Aβ oligomers and aggregates, which are in dissociable equilibrium with the soluble Aβ species. PBT2 reacts with accessible Zn^2+^ and Cu^2+^, promoting dissolution or uptake and degradation of the aggregates. PBT2 also dissociates Zn^2+^ and Cu^2+^ from being trapped by Aβ, neutralizing the charge of the metal ion and allowing it to passively move across cell membranes. This promotes the recycling of Zn^2+^ and Cu^2+^ from the cleft, normalizing functional fluxes, and intracellular metal pools. Aβ, amyloid β; APP, amyloid precursor protein; PBT2, 5,7-dichloro-2-[(dimethylamino)methyl]quinolin-8-ol.

In a small phase 2a double-blind, placebo-controlled, randomized controlled trial (RCT) of PBT2 for AD (n = 29 placebo *versus* n = 29,250 mg/day), PBT2 caused significant executive function *improvement* in only 12 weeks of treatment (70, 71, 72). In other words, PBT2 did not just arrest boosted performance. How could a nootropic benefit from a purportedly disease-modifying drug emerge after only 12 weeks? While both clioquinol and PBT2 were developed to dissipate amyloid pathology through releasing Zn^2+^-bridged Aβ oligomers, this was on the presumption that Aβ aggregates were neurotoxic. A second, smaller, phase 2 RCT of PBT2 used changes in amyloid burden by PiB PET imaging as its primary readout. This exploratory study showed only a trend to decreasing amyloid burden after treatment with PBT2 (250 mg/d, n = 25) compared with placebo (n = 15) for 12 months (73), with no differences in cognitive performance. The study was underpowered for a cognitive readout, and the placebo group cognitive performance did not measurably deteriorate throughout the study (a confound of smaller studies of AD). Thus, the possible nootropic boost of PBT2 in 12 weeks at the first RCT could not be caused by a reduction of amyloid burden. Indeed, the PiB ligand detects fibrillar forms of Aβ, which were never the target of PBT2 (62, 74). As the clinical trials were underway, the mechanism of action of both clioquinol and PBT2 was further investigated, and it became appreciated that these compounds are not high-affinity chelators that lower brain metals, but rather they are copper/zinc ionophores that foster the uptake of Zn^2+^ and Cu^2+^ into cells with notable impact on multiple relevant neurochemical pathways (62, 74, 75). Thus, it became apparent that these ionophores might be therapeutic *not by clearing amyloid* but by normalizing the bioavailability of these essential metal ions otherwise trapped in Aβ aggregates (Fig. 2). The Zn^2+^ released during glutamatergic neurotransmission must be recycled to maintain intracellular stores for various physiological events, including maintaining the expression of the N-methyl-D-aspartate (NMDA) receptor submits. The trapping of Zn^2+^ by extracellular Aβ aggregates can impair neuronal function by causing a deficiency of intracellular Zn^2+^ (33), leading to deficiencies of ProSAP2/Shank3 postsynaptic density assembly (76), deviation of zinc from S100B and NMDA receptor targets (77, 78), as well as interfering with the metabotropic zinc receptor, GPR39 (79). Drug candidates such as clioquinol and PBT2 may act, therefore, to normalize the distribution of Zn^2+^ by facilitating its reuptake and distribution to intracellular targets, as demonstrated in models of autism (80), rather than acting as chelators or reversing Aβ aggregation.

While ZnT3 is responsible for supplying the extracellular Zn^2+^ that promotes extracellular Aβ aggregates, its expression is essential for maintaining cognition with aging, as demonstrated with the accelerated decline in cognition in aging *ZnT3* knockout mice (12). Notably, cortical ZnT3 levels markedly decline with both mouse and human aging and decline even further in AD (12). These changes are associated with decreases in essential components of the synaptic architecture, such as NMDA receptor subunits and PSD95 (12), and are recapitulated in AD, AD models, and neuronal models treated with Aβ, where Zn^2+^ is trapped in the aggregates causing relative intracellular deficiency (33, 76). Decreased cortical ZnT3 levels have also been reported in Parkinson’s disease dementia and Lewy Body Disease (81, 82). Higher levels of ZnT3 were associated with slower antecedent cognitive decline in an unbiased large-scale proteomic analysis of postmortem brain from two tissue banks, even when adjusted for AD pathology (83). Critically, treatment of *ZnT3* knockout mice (with no amyloid) with the zinc/copper ionophore, clioquinol, for 6 weeks corrects the early onset cognitive deficits and normalizes changes in synaptic proteins (84). Similarly, treatment with PBT2 of normal old (22 months) C57Bl6 mice (also without amyloid) restores age-dependent cognitive deficits within 12 days, while rejuvenating synaptic architecture and markers, decreasing phosphorylated tau and significantly increasing zinc (but not copper) in the CA1 hippocampal region but not in any other cortical region (85). The regional selectivity of the zinc elevation in PBT2-treated old mice probably reflects the greater dynamic zinc release and uptake physiology in this region, where zinc turnover is impaired with age (33). These results strongly argue that the benefits of zinc ionophore treatment of amyloid-bearing transgenic mice with clioquinol or PBT2 (62, 67) are mediated by restoring cortical zinc homeostasis and that the amyloid aggregates are a proxy for perturbed zinc trafficking that may exaggerate the problem by trapping more zinc. Therefore, the significant cognitive improvement in a strikingly rapid time frame, 12 weeks, observed in trials (70, 71, 72) is consistent with the time frame in cognitive improvement in each report of these animal models treated with zinc ionophores and therefore most likely reflects the treatment benefits of correcting cortical zinc homeostasis.

Disruption of cortical zinc homeostasis in AD has not been reflected in reports of bulk zinc levels from postmortem tissue (76, 86, 87, 88, 89). However, factors including the accuracy of diagnosis, statistical power, methods of sample preparation, and detection limits may have made changes inconsistent between studies. Also, the total tissue zinc levels might not rise until the plaque burden is severe (57). In the brain of the aged macaque monkey, the difference in zinc concentrations of district brain regions could account for the density of plaques in that region, regardless of the total Aβ level (61). This is reminiscent of the spatial association of plaque burden in *APP* transgenic mice with cortical layers that are most rich in exchangeable zinc (58).

The blood–brain barrier prevents passive fluctuations of plasma zinc from being transduced into the brain. Nevertheless, some reports have explored the impact of dietary zinc on Aβ transgenic models, with inconsistent results reported. Prenatal and postnatal zinc-enriched diets in Tg2576 and TgCRND8 were described to induce accelerated cognitive impairment in these mice (90, 91). Zinc supplementation to *APP*/*PS1* mice was reported to induce Aβ deposition as well as impaired spatial memory (92) but did not affect Tg2576 mice (93). Conversely, dietary zinc deficiency enlarged plaque size in *APP*/*PS1* mice (58). A *drosophila* model of AD overexpressing Aβ was reported to express eye damage that was exaggerated by dietary supplements of zinc or copper but rescued by zinc/copper chelators (94). Nutritional zinc deficiency is common in old age and exacerbates age-dependent cognitive loss in rodents, which can be rescued by zinc supplementation (95). Zinc supplementation to 3xTg-AD mice in adulthood was reported to delay hippocampal-dependent memory deficits and reduce both Aβ and tau (96). However, a meta-analysis of clinical trials of zinc supplementation found no evidence of benefit in treating AD (97).

Changes in the expression of several zinc transporting proteins have also been reported in studies of postmortem brain tissue from AD cases and models (34), although it is difficult to know whether these changes are, like ZnT3, potentially upstream in the pathological process or whether they represent homeostatic responses. The message RNA levels of several ZnT family proteins such as ZnT1, ZnT4, and ZnT6 are increased in AD tissue and correlate with Braak pathological staging (98). ZnT10 was reported reduced in the frontal cortex of AD subjects and *APP*/*PS1* mice (99). The protein level of ZnT6 has been reported to be elevated in the hippocampus/parahippocampal gyrus region of pathologically confirmed AD cases, but the level of ZnT1 was significantly decreased in the same region (100). An inhibitor of cellular zinc export, 4-hydroxynonenal, is induced by lipid peroxidation, a feature of ferroptosis (101). Cytoplasmic-free Zn^2+^ might also be elevated in AD from a depletion of metallothionein III (102, 103, 104), which is the main zinc storage protein in neurons.

Interestingly, zinc may regulate the production of Aβ via affecting the secretases that are responsible for its production. The activity of β-secretase responsible for APP cleavage into the nonamylogenic pathway, a disintegrin and metalloprotease 10, is a zinc metalloproteinase, and mutation of its zinc-binding site abolishes its activity (105). Zinc is also reported to inhibit β-secretase activity *in vitro* (106) and cell culture by induction of APP-C99 fragment dimerization (107), indicating that increased zinc may limit Aβ production. At similar concentrations to those that inhibit β-secretase activity *in vitro*, zinc is also described to promote the production of Aβ43 (107).

There are several reports of zinc interacting also with the other major proteins implicated in AD. Zinc is reported to increase presenilin 1 expression (108) and to affect the stability of apolipoprotein E (ApoE), particularly ApoE4 (109). Conversely, presenilin 1 and ApoE expression have been reported to play essential roles in maintaining cellular and neuronal zinc trafficking (108, 110). Free Zn^2+^ is reported to promote tau phosphorylation and aggregation (Fig. 1). Several kinases and phosphorylases were suggested as mediators of zinc-induced tau hyperphosphorylation in cell culture and mice, including glycogen synthase kinase 3β, cyclin-dependent kinase 5, extracellular signal-regulated kinase, c-Jun N-terminal kinase, and protein phosphatase 2A (PP2A) (111, 112, 113, 114, 115, 116, 117). Alternatively, zinc may facilitate the bridging between Cys-291 and Cys-322 of tau for aggregation, evidenced by point mutation of these sites preventing zinc-induced tau aggregation (118). This may as well lead to toxicity, as zinc can also directly bind to tau protein to promote neurotoxicity independent of hyperphosphorylation in a *drosophila* hTauR406W model (119). Zinc supplementation also has been reported to facilitate the neurodegeneration and tangle formation in P301L mice, a model of tauopathy (120).

The slowing of zinc synaptic turnover with normal aging could lie upstream of the amyloid pathology of AD as well as some facets of cognitive impairment. This warrants more in-depth research, especially because the nootropic benefits of correcting in aging mice without amyloid are provocative and could be the basis of drug interventions for which there is already some clinical trial evidence. The mechanisms for clearing the synapse of zinc released during neurotransmission need to be elaborated urgently. While zinc dyshomeostasis may hamper neuronal function, its connection to neurodegeneration is still unclear, as is the mechanism for synaptic loss in *ZnT3* knockout mice that is corrected by zinc ionophore treatment (12, 62, 74, 84, 85). Insights into this could be very relevant to understanding cognitive dysfunction in AD where there is a marked loss of ZnT3 in cortical tissue despite the accumulation of zinc in plaques (12).

## Copper

**Figure 3:**
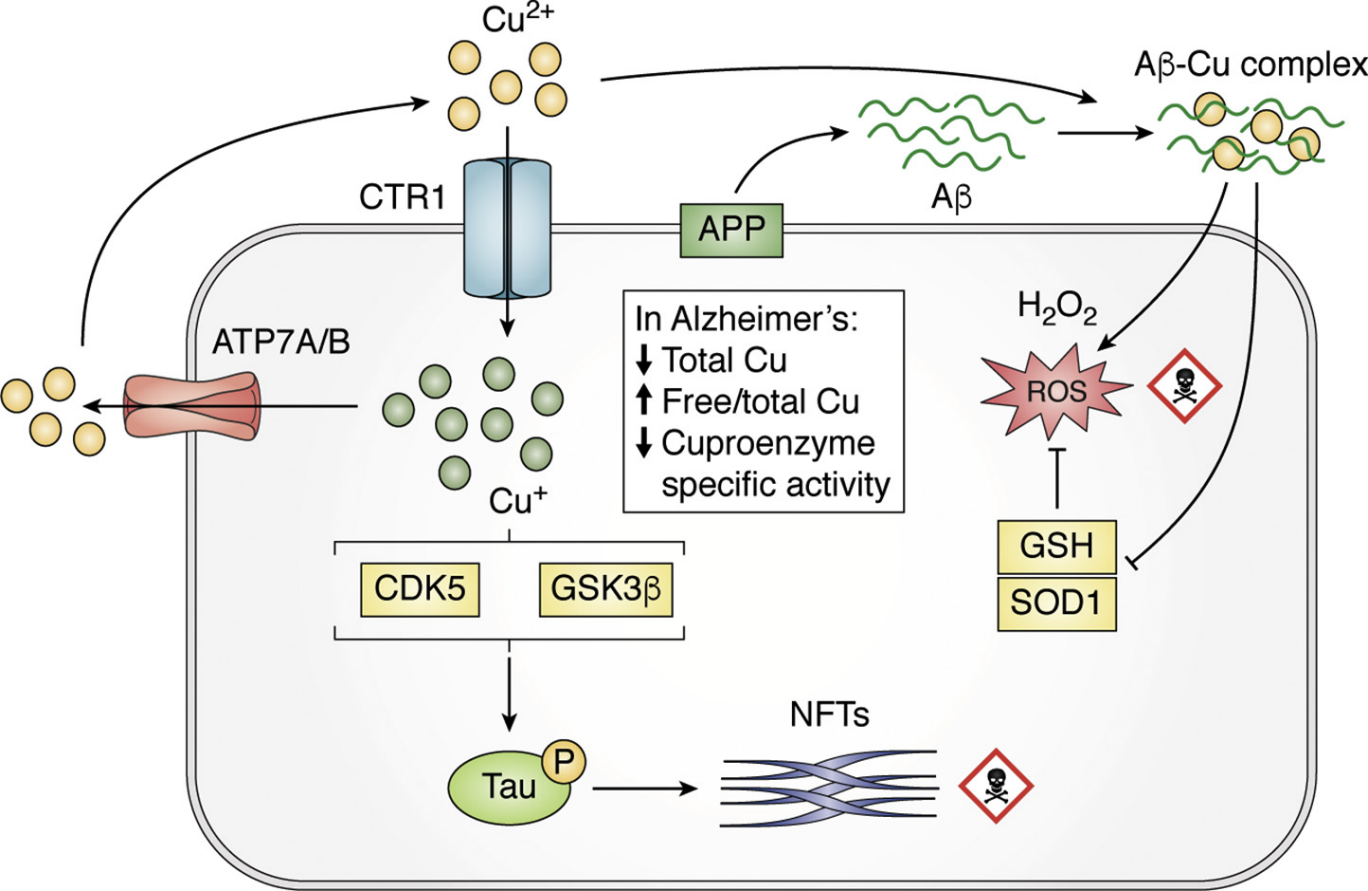
**Copper dysregulation in Alzheimer's disease**. Cu^+^ is taken up into neurons by CTR1 and exported by ATP7A/B. Aβ oligomers can trap extracellular Cu^2+^ and then embed into the membrane, forming a catalytic complex that generates H_2_O_2_. H_2_O_2_ is freely permeable and can migrate to deplete antioxidants like GSH and denature SOD1. In bulk tissue, copper levels are decreased, consistent with a decrease in the activity of ceruloplasmin. But the fraction of cytoplasmic-free Cu^+^ increases in AD-affected tissue, which might contribute to tau hyperphosphorylation by activation of CDK5 or GSK3β. Aβ, amyloid β; AD, Alzheimer's disease; APP, amyloid precursor protein; CDK5, cyclin-dependent kinase 5; GSH, glutathione; GSK3β, glycogen synthase kinase 3β; SOD1, superoxide dismutase 1.

Copper is a redox-active metal that is involved in multiple metabolic activities in the brain, and it serves as the active site for a range of cuproenzymes such as ceruloplasmin (Cp), superoxide dismutase 1 (SOD1), tyrosinase, cytochrome oxidase, etc (121). The regulation of copper transport within all cells is mediated by Ctr1 for uptake and ATP7A/B for efflux (122) (Fig. 3). Mutation of ATP7B (K832R) increases the risk for AD and causes loss of ATP7B function (123). Like zinc, copper concentrations in the synapse elevate transiently during neurotransmission (to 15 μM, from basal levels of ≈0.5 μM based on cerebrospinal fluid (CSF) values [124]), but instead of being released from the bouton, is released postsynaptically upon stimulation of the NMDA receptor (125, 126). Copper can dose-dependently affect LTP, where a low concentration of copper (1 μM) inhibits hippocampal LTP (127), while a high concentration (10 μM) promotes LTP through activation of the α-amino-3-hydroxy-5-methyl-4-isoxazolepropionic acid receptor (128). Several studies have reported that copper supplementation in cultured neurons inhibits the activation of receptors for NMDA, α-amino-3-hydroxy-5-methyl-4-isoxazolepropionic acid, and gamma aminobutyric acid (129, 130, 131). However, the *in vivo* relevance of these findings is yet to be investigated.

Copper concentrations in AD-affected brain tissue are reported to be lower compared with healthy control tissue (132, 133, 134, 135, 136, 137) and accompanied by decreased concentrations of cuproproteins such as SOD1 (138). Despite the decrease in total copper in AD-affected tissue, the proportion of “labile” or loosely bound exchangeable copper ions was increased, indicating a disruption of the average coordination environment of cellular copper ions in the tissue (136). Furthermore, there is evidence that copper concentrates with other metals in amyloid plaques (*vide infra*). Thus, there is a change in the distribution of copper in the AD brain tissue where it is deficient in the cells but trapped in the extracellular plaques. This complex picture is consistent with experimental results, reviewed here, that intracellular copper deficiency promotes Aβ production, whereas extracellular Cu^2+^ pooling can promote Aβ precipitation (under acidic conditions) and oxidative cross-linking and modification. Therefore, neither copper chelation nor copper supplementation are likely to have unopposed benefits, and the theoretical ideal agent would mobilize extracellular Cu^2+^ to be taken back into the cell. Here, we review the evidence for this.

Lowering cellular copper has been shown to increase Aβ production (107, 139, 140, 141, 142, 143), as does a deficiency in the copper chaperone of SOD1 (144). Copper (and zinc) added to human CSF promotes the degradation of Aβ, also consistent with the inverse association between levels of these metals in the CSF and levels of Aβ (124). Earlier studies found that APP can bind to and reduce Cu^2+^ through a site on its N-terminal ectodomain, remote from the Aβ sequence (145, 146, 147, 148). This may subserve a physiological purpose, possibly in copper homeostasis, because APP expression is upregulated by copper (149) and increased APP expression lowers brain, neuronal, and tissue copper levels (150, 151, 152). Also, APP trafficking is sensitive to copper load (139, 153).

In contrast to the background parenchymal brain tissue where copper levels are decreased in AD, amyloid plaques concentrate copper in AD and mouse models of AD (56, 59, 154, 155, 156), supporting the possibility that copper co-aggregates with Aβ (Fig. 3). While APP has separate ectodomain copper (145) and zinc (42, 43) binding sites remote from the Aβ sequence, the copper/zinc binding site in Aβ is overlapping and only emerges once the carboxyl terminus of Aβ is cleaved from full-length APP through the activity of the presenilins. Copper-Aβ interaction was first described in 1994, where Cu^2+^ was observed to strikingly induce soluble dimer formation of Aβ_1–40_ at neutral pH (37), although little precipitation was noted under these conditions (21). Subsequently, Cu^2+^ was found to induce dramatic aggregation of Aβ_1-40_ under mildly physiologically acidic conditions (*e.g.,* pH 6.8) (22) with highest *apparent* affinities of Cu^2+^ for the peptide aggregates being measured as ≈50 pM for Aβ_1-40_ and ≈6 aM for Aβ_1-42_, with the aggregates binding up to three equivalents of Cu per Aβ peptide (38). The very high apparent affinity of Aβ_1-42_ for Cu^2+^ may be a product of the perturbed equilibrium of the peptide–metal complex coming out of solution, but nonetheless the peptide aggregation is reversible with chelation, evidencing proof of principle of pharmacological targeting of the metal center for reversing amyloid formation, which was recapitulated by the solubilization of Aβ from the insoluble fraction of AD-affected brain tissue by copper chelation (24).

These interactions have been extensively studied since (51, 136, 157, 158, 159, 160, 161, 162, 163, 164). It is now understood that Cu^2+^ binds to Aβ residues His6, His13, and His14, and under different pH conditions, Asp1, Ala2, Glu3, and Phe4 can also be involved (165, 166, 167, 168, 169, 170, 171). Mouse Aβ lacks His13 that coordinates Cu^2+^ binding (172) and Zn^2+^ binding (*vide supra*). This is important because mice and rats are exceptional for lacking brain amyloid deposition with age. Zinc (and copper under low pH) induces Aβ oligomerization that favored by greater α-helix content in the peptide. In contrast to metal-free aggregation that proceeds by β-sheet–mediated hydrophobic attraction, zinc-induced aggregation is reversible by dissociating the metal ion (46, 51, 157, 158, 173). Even the trace contaminant metal concentrations (nM) found in neutral buffers is sufficient to promote the seeding and profibrillar aggregation of Aβ peptide solutions and is important to consider in experimental studies (47). Whereas Zn^2+^ induces rapid precipitation of Aβ at neutral pH, Cu^2+^ induces minimal precipitation at neutral pH but profound precipitation under physiologically acidic conditions (pH ≤6.8) (22, 38). The structural basis for this reaction and the pathophysiological significance of this dramatic difference in response to these metal ions has not yet been resolved. Mildly acidic conditions where Cu^2+^ could precipitate Aβ are thought to be present in the synapse, but this view has been challenged (174) and remains to be investigated in AD.

Importantly, copper–Aβ interaction can form a catalytic redox-cycling complex that embeds in lipid membrane and recruits substrates like cholesterol to produce hydrogen peroxide and promote oxidative stress that causes neurotoxicity in cell culture (23, 26, 175, 176, 177, 178, 179, 180, 181, 182, 183, 184, 185). This redox activity is abrogated in the rat/mouse Aβ, which is not only less able to promote the catalytic cycling of Cu^2+^ (and Fe^3+^) but also lacks the Tyr at position 10 (which becomes Phe) to permit dityrosine modification (186, 187, 188). This might also be a factor in rats and mice being protected against amyloid pathology (41). The metal-centered catalytic cycling of human-sequence Aβ in an oxygenated environment not only generates products such as hydrogen peroxide and 4-cholesten-3-one but also oxidizes the side-chains of the peptide, creating dityrosine cross-linked (highly resistant to catabolism) (173, 186, 188, 189, 190, 191, 192) and sulfoxymethionine modification. Dityrosine fragments are enriched in lipofuscin, which is more abundant in AD-affected neurons (193), and, by trapping iron ions, could contribute to the iron burden of the neuron (*vide infra*) (194). The tyrosine (absent in the rodent sequence) and methionine residues of Aβ are reported as critical for toxicity (187, 195, 196, 197). Other posttranslational modifications can inhibit copper–Aβ toxicity by occluding the binding site, *e.g.,* nitration of Aβ (198).

Small model organisms overexpressing Aβ have explored the involvement of copper with amyloid formation, where copper treatment of *Caenorhabditis elegans* overexpressing Aβ in muscle induced Aβ aggregation that is reversed by a copper chelator, and the formation of these aggregates protected the organism against copper toxicity (199, 200). In an Aβ transgenic *drosophila* model, copper chelation therapy, lowering the copper transporter CtrlB or CtrlC, or overexpressing the cellular copper-exporter, DmATP7, reduces the *in vivo* formation of Aβ oligomers and the level of oxidative stress, improving motor deficits and prolonging longevity (201). In the Tg2576 mouse or Aβ cerebral injection model of AD, copper-targeting chelation therapies was reported to improve memory deficits as well as Aβ deposition (202, 203), but the small molecules used often can ligate Zn^2+^, which has not been excluded in these studies. Moreover, wild-type rats, Tg2576 mice, or 3xTg-AD mice with copper-enriched drinking water have been reported to exaggerate cognitive impairment and to worsen neurodegeneration (204, 205, 206, 207), in contrast to one report of dietary copper supplementation suppressing amyloid pathology in transgenic APP23 mice, carrying the Swedish APP mutation (138). For transgenic APP-C100 mice, a model that does not have amyloid deposits, copper exposure had little effect on Aβ production or neuronal survival (208).

Various reports indicate that elevating brain copper levels may suppress amyloid pathology and be therapeutic in AD. Despite the evidence for extracellular copper interacting with amyloid pathology, intracellular copper deficiency has repeatedly been reported to promote amyloidogenesis (107, 139, 140, 141, 142, 143). Measurements of postmortem brain samples from AD cases and AD-transgenic models reveal decreased brain copper levels compared with controls (136, 137), and decreased brain copper has been reported as a feature of the aged healthy human brain (57). Copper depletion increases the generation of Aβ in cell culture (107, 139, 140, 141, 143), and amyloid formation has been reported to be suppressed by intraneuronal copper elevation achieved by genetic modification of APP transgenic mice (209) or by dietary supplementation (138). Based on these findings, a phase 2 randomized clinical trial of copper orotate supplementation in mildly affected AD patients was performed. This 12-months trial revealed no benefit of copper treatment, although there was no readout of target engagement (210). Free ionic copper is unlikely to substantially move from the blood into the brain, especially because 95% of plasma copper is bound to Cp, and the exchangeable fraction is very small. Therefore, maneuvers to deliver copper to the CNS will probably need to involve a chemical chaperone such as the quinolones (clioquinol and PBT2) or the bis(thiosemicarbazone) class.

While most studies have elaborated associations between copper and Aβ or APP, other proteins and pathophysiologies implicated in AD are also reported to interact with copper. Presenilin has been reported to impact on cellular copper turnover and is needed to supply copper for the active site on SOD1 (108, 211). Furthermore, microglial copper trafficking is disturbed via inflammatory responses in the affected cortex of the TgCRND8 (double mutated human APP) transgenic mouse (212). Copper also binds to tau protein *in vitro*, promotes its aggregation, and, similar to the consequence of binding Aβ, can generate hydrogen peroxide (213, 214, 215) by catalytic cycling, as observed for the copper ions bound to neurofibrillary tangles within the neuron in AD affected brain tissue (216) (Fig. 3). Copper can also modulate tau phosphorylation. Copper chelation lowers tau phosphorylation in both cell culture and mice transgenic for tau, with benefits on cognitive function (217). In a triple-transgenic mouse model of AD (mutant APP/PSEN/Tau), copper feeding was reported to increase tau hyperphosphorylation by activating cyclin-dependent kinase 5 (218).

Copper and cuproproteins, including Cp and metallothioneins, have been explored as plasma biomarkers for AD. Plasma total copper levels have been reported as increased in patients with AD, and the non–Cp-bound copper fraction was reported to correlate with the Mini-Mental State Examination scores (219, 220, 221). At variance, data from the Australian Imaging Biomarkers and Lifestyle Study of Ageing (a longitudinal cohort study of 768 cognitive normal elders, as well as mild cognitive impairment and AD cases, with baseline imaging and blood biochemistry, as well as neuropsychological performance assessed at 18-months intervals) indicated that serum non-Cp–bound copper might be decreased (222). In the CSF, however, Cp levels were reported not to be elevated in AD but were associated with CSF ApoE levels, longitudinal cognitive decline, and brain volume loss (223).

The bis(thiosemicarbazone) scaffold coordinates Cu^2+^ with a range of affinities, depending on its side groups. Members of this chemical class have been considered as potential PET imaging agents that could exchange radiocopper with Aβ as a guide to Aβ deposition (224, 225, 226, 227). Two of these, CuGTSM and CuATSM, have been tested in animal models of AD. CuGTSM has a much lower affinity for Cu^2+^ compared with CuATSM yet was far more potent than CuATSM in rescuing cognitive impairment in *APP*/*PS1* transgenic mouse model (228). This benefit may be because of more dissociation of Cu after cellular uptake, with a consequent impact on glycogen synthase kinase 3β activity (228). PET imaging using ^64^CuGTSM and ^64^CuATSM revealed markedly more ^64^Cu uptake into the brains of an *APP*/*PS1* transgenic mouse model compared with wild-type controls for ^64^CuGTSM but not for ^64^CuATSM. Additionally, treatment of AD brain sections showed no binding to amyloid plaques (229). A further report of ^64^CuGTSM confirmed that there was greater uptake into the brains of PS1/APP transgenic mice compared with wild-type controls, yet the brain regions with the highest density of amyloid showed the lowest accumulation (230, 231). There was far less uptake of ^64^Cu-acetate than ^64^CuGTSM into the brains of either normal or PS1/APP mice, but this uptake was greater in younger mice (231). Taken together, these data underscore the impression from the studies reviewed above that supplementing the brain uptake of copper may be beneficial in AD by correcting a deficiency in brain copper that arises from age or from pathology. Achieving this therapeutic copper supplementation requires an ionophoric scaffold (*e.g.*, GTSM, PBT2, clioquinol). Although CuATSM did not benefit the *APP*/*PS1* mouse model, its PET radioligand detects neurodegeneration in Parkinson’s disease and amyotrophic lateral sclerosis patients, and recently, CuATSM (but not CuGTSM) has been reported to interact with the lipid peroxyl groups that are formed during ferroptosis. This is mediated through CuATSM aryl amines rather than through the exchange of free ionic copper (232). This molecule did not exchange radiocopper with plaques in an animal model of AD, possibly because of its attomolar affinity for Cu^2+^, yet as a PET ligand could be useful for detecting ferroptosis if this form of cell death occurs in AD.

Clioquinol and PBT2 target the metal ion in copper–Aβ complexes (as well as zinc–Aβ complexes), rescue preclinical animal models, and have been trialed in patients. Both clioquinol and PBT2 are ionophores that promote the uptake of both Zn^2+^ and Cu^2+^. It is therefore uncertain whether the exact biochemical target is copper or zinc, although zinc is more abundant (150 μM) than copper (15 μM) in neocortex and both clioquinol and PBT2 rescue cognitive loss in aging ZnT3 knockout mice as well as aged normal mice (84, 85). Copper-containing bis(thiosemicarbazone) ligands, which also act as ionophores, have been shown to transport copper into the neuron, lower Aβ levels in both cell culture and animal models of AD, promote neurite elongation, and rescue the cognitive deficits observed in *APP*/*PS1* mice (228, 233, 234, 235). Although there is no AD trial yet for this class of compounds (*e.g.,* CuGTSM and CuATSM), these compounds have been shown beneficial in animal models of several neurological disorders (236, 237, 238). CuATSM has also been reported to induce favorable outcomes in phase 1 clinical trials of Amyotrophic Lateral Sclerosis (NCT02870634) and Parkinson’s disease (NCT03204929). Taken together, liberating zinc and copper trapped by amyloid (or tangles) or promoting the uptake of these metal ions into the tissue could have several beneficial effects in AD, and on the available evidence, it is difficult to attribute the reported benefits to either metal ion. Whereas the impaired turnover of extracellular zinc and copper could likely contribute to amyloid formation and neurophysiological dysfunction, it is not yet clear how this could propel neurodegeneration, in contrast to iron, where a form of regulated cell lethality, ferroptosis, could be at play.

## Iron

Iron is the most abundant transition metal in the brain, and it participates in essential and diverse brain activities, such as the synthesis of neurotransmitters, myelination, and mitochondrial function (239, 240). Recently, iron was discovered to translocate among brain regions along with specific axonal projections: a pathway from the ventral hippocampus to the medial prefrontal cortex to substantia nigra, and a pathway from thalamus to the amygdala to the medial prefrontal cortex. Iron translocation in components of this pathway was shown to modulate anxiety behavior (241, 242), but the discovery opens a new trafficking mechanism where other pathways may exist and be perturbed in other brain disorders such as AD. Like ionic copper, iron changes valence state in biochemistry between the ferrous Fe^2+^ and the ferric Fe^3+^ species. While this property is vital in physiology, it can also be deleterious as a source of oxidative stress, especially in an obligate aerobic environment. Thus, iron is tightly regulated in the brain, where both deficiency and overload of iron may cause dysfunction of the brain (239). Iron deficiency delays neurodevelopment in early life stages (243). Conversely, age-dependent iron accumulation in the brain is an invariable consequence of aging and may contribute to several neurodegenerative disorders, including AD (244, 245). The cause of brain iron accumulation with aging is uncertain. Cellular iron sequestration is a canonical feature of inflammation (246), and inflammatory changes are more prevalent in the brain with aging. Lipofuscin (“aging pigment”) accumulates adjacent to mitochondria in aging neurons, and whereas its pathophysiological significance is unclear, and it contains very high concentrations of iron and other metal ions coordinated by oxidized peptide fragments (194). This pool might also adversely feed the burden of iron in the brain tissue.

Postmortem examinations of AD brains with advanced technology such as laser ablation-inductively coupled plasma-mass spectrometry imaging have revealed that iron accumulates explicitly in the frontal cortex and hippocampus, areas that are most affected by AD proteinopathies (86, 247, 248, 249, 250, 251). Laser ablation-inductively coupled plasma-mass spectrometry reveals not only the concentrations of iron in locations at the subcellular level but co-localizes the concentration of iron in the pixel with the concentration of a protein target assayed by an antibody labeled with a rare-metal (*e.g.,* Au) (251, 252). Reports of elevated postmortem tissue iron are inconsistent. Insufficient detection limits, small sample sizes in early studies, inaccuracies in the clinicopathological diagnosis of AD, and iron-depletion by fixatives may have contributed to this variance (88, 253, 254). A large recent study described that iron accumulation in the inferior temporal cortex could only be found in subjects both diagnosed clinically for AD and confirmed postmortem by standardized criteria (255). Such confirmation of diagnosis was missing in earlier studies.

In contrast to postmortem values, *in vivo* assessments of brain iron levels by magnetic resonance imaging (MRI) have consistently detected iron elevation in the AD-affected brain. The signal attributed to iron detected by MRI techniques (*e.g.,* relaxometry and quantitative susceptibility mapping [QSM]) differs to the iron measured by destructive techniques (*e.g.,* ICP-MS or furnace spectroscopy). The destructive techniques quantify total iron content, whereas the iron detected by MRI is contextual and modified by regional magnetic and conduction properties of the tissue (*e.g.,* myelin). So, MRI changes for iron signals may reflect both qualitative and quantitative changes. Nevertheless, tandem pathology has validated that MRI detects an abnormally elevated pool of iron in AD-affected brain tissue (249, 250, 256, 257). QSM is believed to be the most selective MRI modality for tissue iron (258). By MRI, significantly elevated iron signals have been reported in the bilateral hippocampus, parietal cortex, frontal white matter, putamen, caudate nucleus, and dentate nucleus of AD patients compared with healthy controls (256, 259, 260, 261, 262). A significant negative association between age and entorhinal cortex volume was only present in individuals with both elevated Aβ by positron emission tomography and iron by MRI (263). Using QSM, iron accumulation in AD was found to be associated with cognitive impairment, brain atrophy, Aβ deposition, and tangle deposition (256, 264, 265, 266, 267, 268), confirming the possible utility of iron as a biomarker for AD progression.

Similar associations have also been reported using CSF ferritin as a proxy for brain iron burden. Baseline CSF ferritin was reported to strongly predict cognitive deterioration over 7 years in the AD Neuroimaging Initiative cohort (269). Additional analysis of AD Neuroimaging Initiative data revealed that CSF ferritin strongly associated with cognitive decline in *APOE-ε4* carriers compared with noncarriers (270); higher baseline CSF ferritin predicted accelerated transition from a normal level of CSF Aβ to a level meeting biomarker criteria for AD (271); furthermore, CSF ferritin also interacts with CSF total-tau/Aβ_1-42_ ratio to predict brain hypometabolism (reporting reduced brain function and neurodegeneration) (272). Only 9% of the variance in CSF ferritin could be explained by plasma ferritin, indicating that there is not much exchange of ferritin between peripheral and central compartments (269). Nonetheless, plasma ferritin was reported to be more commonly abnormally elevated in AD patients from the Australian Imaging Biomarkers and Lifestyle Study of Ageing cohort (273) and has been reported as increased in subjects with a high neocortical Aβ load (274). A similar increase in CSF ferritin was recently reported in the BioFinder cohort (246). AD individuals also have lower hemoglobin as well as decreased plasma iron levels, indicating a possible disturbance of peripheral iron metabolism in AD (273, 275).

Consistent with the association of levels of iron in brain tissue with neurodegeneration, considerable evidence indicates that the stringent regulation of brain iron homeostasis has broken down in AD. Recent unbiased single-cell transcriptomics and proteomic analyses confirmed that iron pathways are prominently perturbed in AD brain tissue (276, 277). Genetic studies of the canonical iron regulating genes have revealed an influence on the risk for AD. The coding polymorphism Pro570Ser of *TF* (the gene encoding transferrin, the major protein responsible for iron supply) has been reported to increase AD risk with an odds ratio of 1.21 (278). High ferrum coding polymorphisms (H63D and C82Y) are independent risk factors for AD (279, 280). The major genetic risk for AD is the *APOE*-ε4 variant, which has been reported to elevate brain iron levels (269). Recent studies have reported that the canonical iron-associated genes interact with the *APOE*-ε4 risk allele and further increase AD risk (281, 282, 283). Transferrin protein levels have been reported as elevated in AD frontal cortex (284), and Cp, which facilitates cellular iron export, has been reported as downregulated in AD brain tissue (285, 286, 287). Hepcidin, which destabilizes the iron-export protein ferroportin, as well as ferroportin itself, is also reported to be down-regulated in AD cortical tissue (288). Although the decrease in ferroportin could account for a local rise in cellular iron, the drop in ferroportin could not have been caused by the action of hepcidin, because hepcidin levels were decreased. Therefore, the cause of these changes is uncertain. Hepcidin is expressed in many brain cells, and its role in brain homeostasis is complex because it is expressed at the blood–brain barrier, and its high expression in astrocytes can indirectly influence iron homeostasis in neurons (289). Indeed, adeno-associated virus mediated overexpression of hepcidin in astrocytes rescued cognition and neuropathology in the *APP*/*PS1* transgenic mouse model for AD (290).

**Figure 4:**
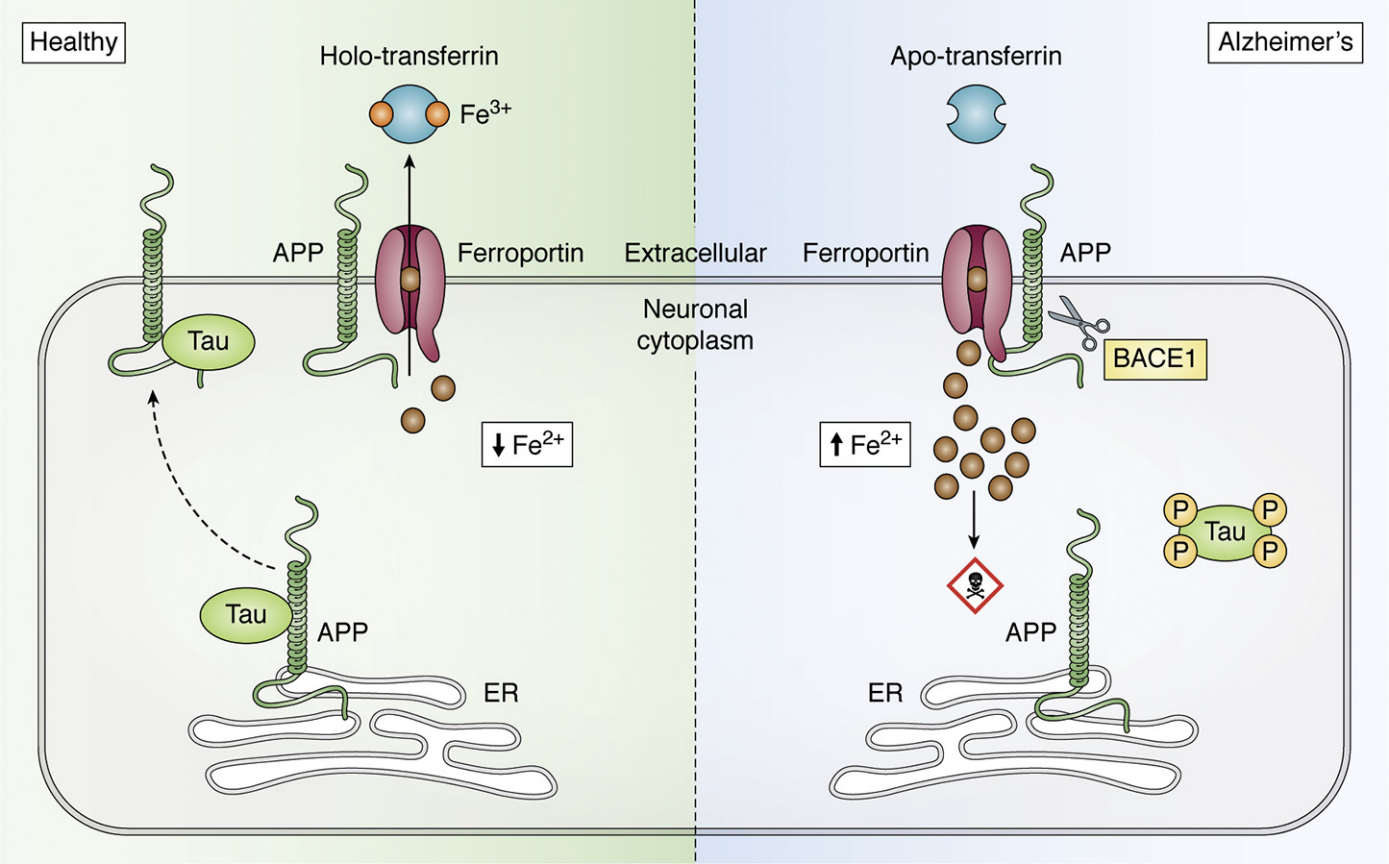
**AD-associated proteins and iron transport.** In health, neuronal iron (Fe^2+^) export is regulated by tau-mediated APP trafficking. Tau guides the trafficking of APP cargo to the neuronal surface, where APP interacts with and stabilizes ferroportin, facilitating iron export from neurons. A reduction of soluble tau or FAD mutation of APP impairs iron export from neurons and results in iron retention. Similarly, when APP is cleaved by BACE1, ferroportin is not stabilized on the surface and does not function to export iron. The intracellular accumulation of Fe^2+^ increases the susceptibility to ferroptosis. AD, Alzheimer's disease; APP, amyloid precursor protein; BACE1, β-site amyloid precursor protein cleaving enzyme 1; FAD, familial AD.

The proteins that form AD proteinopathy, APP and tau (291), have been linked to iron metabolism (Fig. 4). An iron-responsive element in the 5’-untranslated region of the APP transcript promotes the translation of APP in response to iron challenge (292, 293, 294). Iron has also been described to promote the α-processing of APP (295, 296, 297), which may relate to how the divalent metal transporter 1 is reported to promote processing (298). Additionally, β-site amyloid precursor protein cleaving enzyme 1 (BACE1) activity is inhibited by Fe^3+^ with an IC_50_ of 22 μM (297), a concentration potentially achievable in an endocytic compartment, where BACE1 cleaves APP. Conversely, APP protein was reported to promote the export of neuronal iron by stabilizing surface ferroportin, the obligate iron export transporter (299). This resembled the activity of the ferroxidase Cp in promoting the export of iron from nonneuronal cells by stabilizing ferroportin and promoting the loading of iron into transferrin through oxidation of ferrous iron (300). Initially, it was thought that APP itself possessed ferroxidase activity because it possessed a site with homology to the ferroxidase catalytic site H-ferritin, but the oxidation measured was an artifact of contaminating phosphate buffer from the purification of the APP. Nonetheless, APP has been consistently reported to stabilize surface ferroportin, supporting a specific role in facilitating iron export from neurons (301, 302, 303, 304). Consistent with this proposed function, primary neuronal cultures from APP knockout mice retained iron compared with wild-type neurons (11, 299). Overexpression of APP in SH-SY5Y neuroblastoma cells, and the overexpression of the C-terminal 100 residue fragment of APP in the brains of transgenic mice, lowered iron levels (305, 306). Site-directed mutation of the N-glycosylation (N467K and N496K) or ectodomain phosphorylation (S206A) sites, close to the ferroportin binding site on APP, resulted in decreased cell-surface stabilization of ferroportin and consequent iron accumulation (303).

Recently, amyloidogenic processing of APP was found to impact on iron export through stabilizing surface ferroportin (304). Two mutations of APP were studied—the pathogenic Italian mutation A673V and the protective Icelandic mutation A673T. These mutants of the same site of APP were thought to induce or protect against AD by biasing the processing of APP toward or away from β-secretase cleavage, respectively, so promoting or inhibiting the generation of Aβ. However, the Italian mutation that generates more sAPPβ also fails to stabilize ferroportin on the neuronal surface, inducing iron retention. In contrast, the favorable Icelandic mutation generates more sAPPα and, by stabilizing more ferroportin on the neuronal surface, promotes greater iron export than wild-type APP. These findings were recapitulated by pharmacological inhibition of α-secretase and β-secretase processing, respectively, and by other pharmacological approaches that modulated endocytotic processing pathways accordingly (304). Thus, these pathogenic or protective APP mutations might induce or protect against neurodegeneration through their impacts on neuronal iron retention, in which case the generation of Aβ is circumstantial rather than causative. Whether this potential mechanism indeed promotes neuronal death (*e.g.,* through ferroptosis) and whether it occurs in other FAD mutations are currently areas of active investigation. However, these findings support the possibility of antagonistic pleiotropy, where the FAD mutation has advantages in early life by promoting the retention of essential neuronal iron under a geographic condition where nutritional iron is limiting. In contrast, in late-life, this iron burden is a liability. A nonamyloid explanation for AD-causing genetic mutations should be a welcome innovation.

Another physiological intersection between APP and iron homeostasis is with its interaction with heme oxygenase 1 (HO-1), an intracellular enzyme responsible for the breakdown of heme into free Fe^2+^, CO, and biliverdin. Although HO-1 can reduce oxidative stress by lowering the burden of pro-oxidant heme, overactivity of HO-1 can present excess Fe^2+^ to the cytoplasm and induce oxidative stress and promote ferroptosis. Increased HO-1 has been consistently reported to be increased in astrocytes in AD-affected brain tissue but decreased in plasma and CSF (reviewed [307]). Intriguingly, APP has been reported to inhibit HO-1 and HO-2, with the FAD mutant APP species binding with higher affinity (308, 309, 310).

Tau protein also has been described to act in concert with APP to promote iron export. Our group has described that tau mediates the trafficking of APP to the cell surface where APP promotes iron efflux by stabilizing ferroportin; thus, tau ablation significantly attenuates iron transport *in vitro* and *in vivo* (9, 311, 312, 313). Soluble tau is reported to be reduced in Alzheimer’s patients (314, 315, 316, 317, 318, 319), and such loss in mice causes iron accumulation and, consequently, neurodegeneration, which can be rescued by iron chelation or antioxidant supplementation (9, 320, 321). We and others reported that pharmacologically suppressing tau expression with lithium (322, 323) caused APP- and tau-dependent iron accumulation, and indeed the treatment of human subjects with lithium increased iron in their hippocampus and substantial nigra (312, 324). The ubiquitously expressed phosphatidylinositol binding clathrin assembly protein, identified in genome-wide association studies for late-onset AD, also regulates iron uptake, and its suppression renders the cell more sensitive to iron chelation (325). Interestingly, iron can also regulate the expression of *APOE* at the posttranscriptional and transcriptional levels in both neurons and astrocytes, increasing its secretion (326). Collectively, these results indicate that dyshomeostasis of iron is associated with the proteins most implicated in AD pathology.

**Figure 5:**
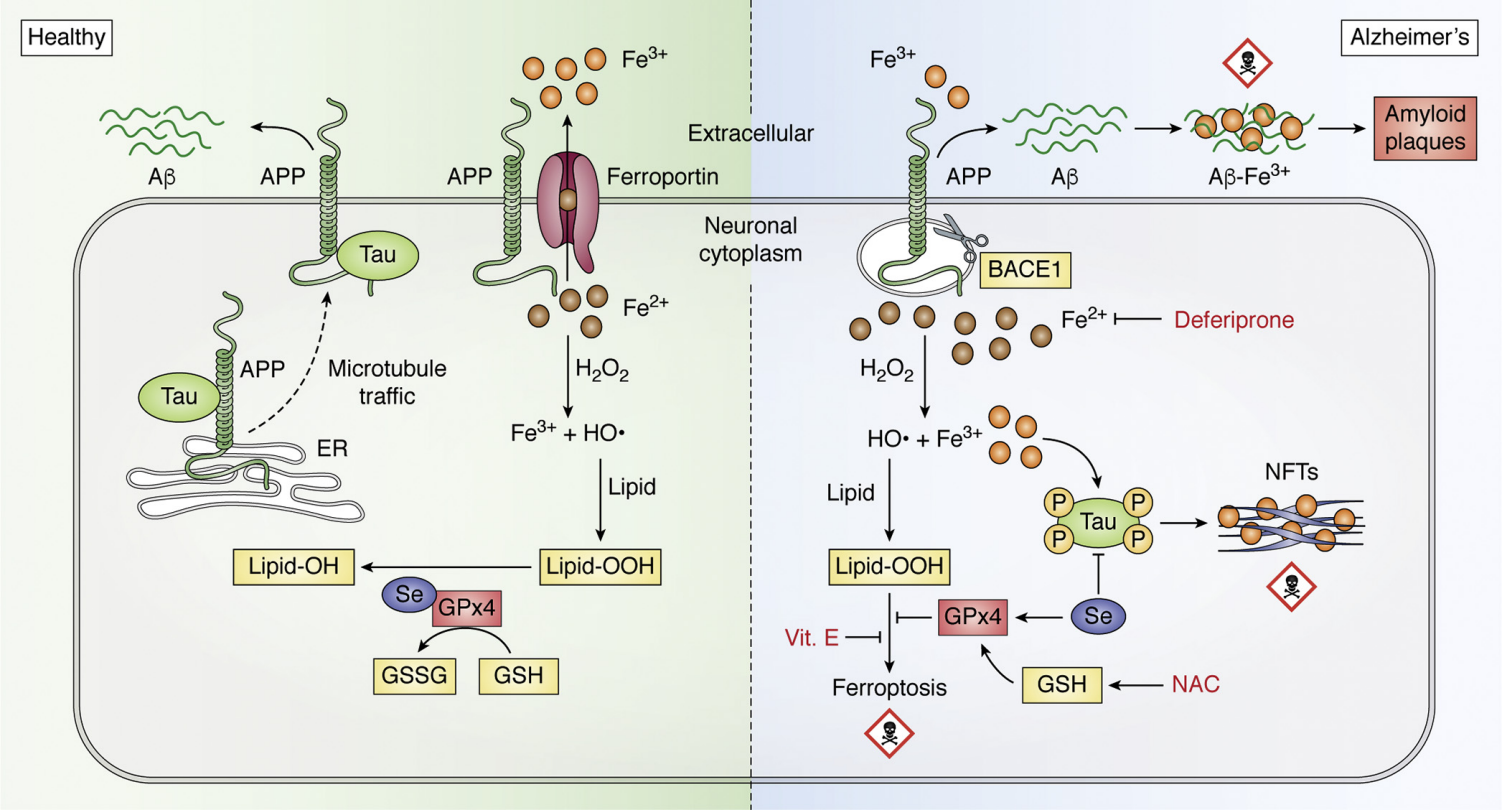
**Ferroptosis in Alzheimer's disease.** In health, selenium (Se) in the brain can inhibit Aβ generation and tau hyperphosphorylation by modulating PP2A activity. Selenocysteine promotes the synthesis of GPx4, where it forms the active site. Accumulation of iron (*e.g.,* from aging or from BACE1 processing of APP) increases the reaction of cytoplasmic Fe^2+^ with H_2_O_2_ to generate the hydroxyl radical (HO, Fenton chemistry), which then reacts with PUFA-containing membrane phospholipids, generating lipid peroxides and initiating lipid radical propagation, which then disrupts the plasma membrane and causes ferroptosis. This mechanism is initiated by autoxidation, but arachidonate lipoxygenase 15-mediated peroxidation of phospholipids can also initiate ferroptosis in an iron-dependent manner (374). Se, N-acetylcysteine (NAC), α-tocopherol (Vit E), and deferiprone act on different components of the pathway to prevent ferroptosis, potentially accounting for their putative clinical benefits for AD. AD, Alzheimer's disease; Aβ, amyloid β; APP, amyloid precursor protein; BACE1, β-site amyloid precursor protein cleaving enzyme 1; GPx4, glutathione peroxidase 4; PP2A, protein phosphatase 2A; PUFA, polyunsaturated fatty acids.

In AD iron could contribute to pathology in several ways (Fig. 5). Iron might drive the formation of plaques and tangles, evidenced by elevated concentrations of iron in the senile plaques (256, 327, 328) and co-localization with tangles (329). In cell-free systems, iron promotes Aβ aggregation, promoting neuronal toxicity (330, 331, 332, 333, 334, 335). The toxicity is most likely from Fenton chemistry (25, 175, 336). Iron is reduced by Aβ peptides, which fosters thiobarbituric acid substances reactivity greatest when generated by Aβ_1-42_, Aβ_1-40_ > rat Aβ_1-40_, in accordance with their participation in amyloid pathology. The toxicity of Aβ–iron complexes might also be because of the specific structure of Aβ aggregation induced (334), which is prone to activate cell death pathways (337). The binding site of Aβ for iron includes His6, His13, and His14 (175, 338, 339) but overlaps with the residues that coordinate Zn^2+^ and Cu^2+^, and in the rat/mouse, sequence is substituted R5G, Y10F, H13R. The tyrosine substitution may also attenuate the redox activity of the peptide associated with its toxicity (187). Similarly, iron can bind to tau (340, 341) and facilitate tau aggregation (342) and hyperphosphorylation (343, 344, 345), which can be reversed by iron chelation (344, 346). Phosphate groups coordinate Fe^3+^ with very high affinity and hyperphosphorylated tau can be purified from postmortem AD brain samples on the basis of this affinity (347). This indicates that while phosphorylation of tau dissociates tau from microtubules, elevated cytosolic Fe^3+^ as occurs in aging, and AD might neutralize the charge on the phosphates and promote aggregation. Alternatively, that a large collection of hyperphosphorylated tau could inappropriately ligate cytoplasmic Fe^3+^ is a reasonable hypothesis that remains untested. Finally, the gliosis that characterizes AD pathology may also contribute to deleterious iron-mediated reactions (348, 349).

Taken together, these findings build a case for how iron, either building up in the tissue, bound to the amyloid or tangle proteinopathy, inducing the proteinopathy, or in tandem with the proteinopathy, might contribute to AD pathophysiology. Targeting iron, therefore, might be a therapeutic strategy for AD. Animal studies of deferoxamine (DFO), a potent iron chelator, was reported to rescue memory deficits, inhibiting amyloidogenic APP processing and Aβ aggregation (350, 351, 352). Other iron chelators have been developed and tested in animal models of AD, with beneficial results for neurodegeneration (353, 354, 355, 356). In 1991, a report of a 2 year, single-blind study of 48 patients investigated intramuscular DFO as a treatment for AD. Although the investigators theorized that DFO would chelate aluminum, DFO is more potent chelating iron than aluminum, and iron is 1000-fold more abundant than aluminum in the brain tissue. The results of this single-blind phase 2 study indicated a substantial reduction in the rate of deterioration of daily living skills associated with DFO treatment (357). This trial was never followed up. The orally bioavailable brain permeable iron chelator, deferiprone, lowers brain iron in children with mutant pantothenate kinase neurodegeneration (Neurodegeneration with brain iron accumulation type 1) (358, 359) and in adults with Parkinson’s disease where reported clinical benefits in phase 2 studies (360, 361) are currently being tested in phase 3 in Europe (FAIRPARK-II, NCT02655315). We are currently testing the same dose of deferiprone in a randomized, multicenter, double-blind, placebo-controlled phase 2a trial for AD (Deferiprone to Delay Dementia, the 3D study, NCT03234686).

## Ferroptosis and selenium

The mechanism of neuronal death in AD is still uncertain. With considerable evidence circumstantially implicating iron burden in the pathogenesis of AD, a role for iron proximal to neuronal demise has been underscored by the elaboration of ferroptosis—an iron-dependent, nonapoptotic form of regulated cell death, which is mediated by the propagation of excess lipid hydroperoxides (362). First described in 2012, ferroptosis and its regulation have rapidly garnered interest for potential roles in various cell death events (363, 364, 365, 366). AD-affected brain tissue exhibits many pathological changes that are consistent with ferroptosis (363). Because the iron burden is associated with neurodegeneration and clinical deterioration in AD, we consider here whether ferroptosis could contribute to neurodegeneration in AD through the actions of iron itself and the Se-dependent checkpoint for ferroptosis, GPx4 (Fig. 5).

The oxidation of lipids (polyunsaturated fatty acids, PUFAs) by iron initiates ferroptosis, but this is not usually a consequence of elevated cellular iron (although the probability of lipid peroxidation rises with iron burden). The cytoplasmic free Fe^2+^ concentration is ≈2 μM, and is kept stable by a variety of mechanisms including buffering by cytoplasmic storage in redox-silent ferritin cages where a 24-mer can hold ≈4000 iron atoms in an oxidized state (reviewed [367]). However, the cytoplasmic free Fe^2+^ concentration is high enough to react with PUFAs constantly, and therefore, there is a tonic clearance of the lipid peroxides that form to forestall inappropriate ferroptosis. This is largely the function of the checkpoint enzyme, GPx4 (363, 368, 369). The selenocysteine active site of GPx4 (368) cannot be adequately substituted, *e.g.*, with cysteine (369). GPx4 converts the toxic phospholipid hydroperoxides (lipid-OOH) to nontoxic phospholipid alcohols (lipid-OH), utilizing the electron donated by glutathione (GSH), generating oxidized GSSG as a by-product (370). Fe^2+^ can also bind to GSH, which stabilizes its ferrous state, and prevents it from participating in ROS generation (371). The levels of cytoplasmic GSH can play a critical role in initiating ferroptosis. The synthesis of GSH requires glutamate and cysteine (the rate-limiting substrate), which is a reduced product of cystine. Both cystine and glutamate are transported across the plasma membrane by System ${\text{X}}_{c}^{\;–}$ (a glutamate-cystine antiporter) and excitatory amino acid transporters, respectively (372, 373). The most common methods for inducing ferroptosis *in vitro* are with small molecules that deplete GSH (*e.g.,* erastin that blocks the ${\text{X}}_{c}^{\;–}$ antiporter, depleting the cell of cysteine for GSH synthesis) or inhibit GPx4 (*e.g.,* RSL3, which covalently binds to the selenocysteine active site) (363, 365, 374). Direct administration of iron to cells in culture, or indirectly promoting iron influx by adding transferrin, facilitates erastin-induced ferroptosis (362, 375).

The mechanism(s) by which iron executes ferroptotic cell death is debated. It is proposed that cytoplasmic Fe^2+^ directly reacts with membrane lipids, triggering a lethal lipid radical chain reaction and pore formation (374). Rescue by osmoprotectants supports this likelihood (376). There are a number of means of aborting ferroptosis (363), including upstream intervention with N-acetylcysteine (NAC, a precursor for glutathione) or iron chelation and downstream intervention by neutralization of lipid radicals with radical trapping agents (RTAs). Small molecule RTAs include α-tocopherol (vitamin E, with an IC50 at high μM–mM concentrations) or highly potent organic molecules (liproxstatin-1 and ferrostatin-1, with IC50s in the low nM range). This class of very high potency RTAs is selective for inhibiting ferroptosis among the forms of cell death. Intriguingly, CuATSM (*vide supra*) was recently reported to be a high potency RTA-class ferroptosis inhibitor (232). This could be the mechanism of CuATSM rescuing mouse models of the neurodegenerative diseases, Parkinson’s disease and ALS. Phase 1 results for these indications were favorable, and if the current phase 2 testing for ALS is successful, CuATSM may be worth trialing in AD.

Iron can also signal other relevant prodeath pathways that may occur separately or in tandem with ferroptosis. Iron loads hypoxia-inducible factor prolyl hydroxylases that activate ATF4-dependent prodeath transcription (377). Also, iron-dependent arachidonate lipoxygenase enzymes facilitate lipid peroxidation of plasma membrane PUFAs (378), ultimately still leading to ferroptotic-type cell death that is mediated by plasma membrane osmotic opening that is propagated but rescued by high potency RTAs (376). In cell culture, treatment with iron by itself only induces cell death upon challenge with high concentrations (mM range), in contrast to potent ferroptosis inducers erastin and RSL3 (nM–low μM range). Nonetheless, iron loading of tissue increases sensitivity toward ferroptotic signals, as recently demonstrated by the impact of iron chelation or liproxstatin-1 treatment doubling the lifespan of the *C. elegans* aging model without adversely affecting metabolism or healthspan (379).

*GPx4* homozygous knockout (*Gpx4*^−/−^) is embryonically lethal in mice (E7.5) (380, 381), and neonatally lethal in neuron-specific *GPx4* knockout mice (382). Inducing somatic *GPx4* knockout in mice at 6 to 9 months of age results in an aggressive neurodegeneration phenotype, hippocampal neuronal death, and demise within 2 weeks of onset, underscoring the importance of GPx4 in neuronal viability (383). An inducible- and regional-specific knockout of GPx4 in the forebrain results in forebrain neurodegeneration and cognitive deficits (384).

In AD, both GSH and GPx expression have been reported to be significantly downregulated in the frontal cortex and hippocampus and correlated with the severity of the impairment (385, 386, 387). Excitatory amino acid transporters 2 and 3 were also found to be reduced in the hippocampus in AD (388). This evidence indicates that the GSH pathway is compromised in AD, promoting lipid peroxidation (389), the executioner of ferroptosis. Similarly, GSH depletion was also observed both in cell culture and animal models of AD (390, 391). The guanine-rich RNA sequence binding factor 1 that controls GPx4 translation has been reported to be downregulated in a mouse model of AD that expresses brain lipid peroxidation (392). Thus, both iron accumulation and a compromised GSH pathway foster an environment to promote ferroptosis in AD (Fig. 5).

A number of compounds have been evaluated in AD models that later emerged as ferroptosis inhibitors. For example, NAC treatment of mice impaired by intracerebral injection of Aβ increased GSH content and suppressed lipid oxidation and rescued cognitive deficits (393). A small (n = 23 NAC, n = 20 placebo) randomized, double-blind, placebo-controlled phase 2 clinical trial of NAC (50 mg/kg/d in 3 divided doses) over 6 months for the treatment of AD revealed overall benefits and significantly arrested deterioration on several cognitive tests (394) but has never been followed up on a larger scale. Treatment of P301S tau transgenic mice with α-lipoic acid improved memory and cognition compared with control-fed mice while increasing GPx4 expression and mitigating signs of ferroptosis in the brain (395). α-tocopherol (Vitamin E), a lipid radical scavenger and a low-potency ferroptosis inhibitor, can protect neurons in rats injected with iron (396). This may be the mechanism of benefit of α-tocopherol in a phase 3 clinical trial in AD, where the supplement at 2000 IU/d significantly delayed functional decline (where the prescription drug for AD, memantine, did not) (397).

As mentioned earlier, Se plays a central role in ferroptosis through its essential role as the active site of GPx4. While selenocysteine is the essential 21st amino acid incorporated into selenoproteins, its production can be boosted by supplementing Se in various forms including organic (*e.g.,* selenomethionine) or inorganic (*e.g.,* selenite, selenate) pharmacological species (368). Selenopeptides have been shown to increase GPx4 expression, so protecting neurons against ferroptosis in animal models of hemorrhagic and ischemic stroke (398).

Se has long been implicated in AD pathogenesis (Fig. 5). Plasma Se has been reported as lower in AD patients compared with healthy elderly, according to two small cohort studies (399, 400) but unchanged in serum or CSF in another AD cohort (401). A decrease in the affected temporal cortex of AD brains (402) has been confirmed by meta-analysis (403), and the Se content in the brain is reported as lower in *APOE*-ε4 carriers (402).

Se rescues the streptozotocin-induced rat model of cognitive impairment (404). Directly applying Se to cell culture results in reduced Aβ production by lowering BACE1 and protects against Aβ toxicity while lowering 4-hydroxynonenal, a downstream marker of ferroptosis (405). Overexpressing Selenoprotein M, or supplementation with selenomethionine, or the lipid-soluble Se compound ebselen, also lowered Aβ production (406, 407, 408, 409, 410). These studies highlight the possibility of ferroptosis involvement in Aβ pathophysiology that had been previously unappreciated. One possibility is that Aβ generation is a response to or an epiphenomenon of brain ferroptosis. Consistent with this possibility, dietary Se deficiency caused a two-fold increase in plaque deposition in Tg2576 mice (411).

Similarly, there is evidence that links tau pathology to ferroptosis. Sodium selenite has been shown to reduce tau phosphorylation *in vitro* and *in vivo* through activation of serine/threonine-specific PP2A, and it rescues cognitive deficits in tau transgenic mice including P301L, K369I, and TAU441 models (406, 412, 413, 414). Se-Met rescued cognitive deficits while normalizing synaptic proteins and lowered phosphorylated tau through PP2A activation in 3xTg AD mice (415). Alternatively, supplementation of Se increases the expression of Selenoprotein S, which mitigates ER stress, and is co-localized with tangles in AD brains (416).

A great deal more experimental evidence is needed to test the possibility that the proteinopathies of AD are associated with underlying ferroptosis. The proteinopathies have traditionally been regarded as the cause of neurotoxicity, but the possibility that the true toxicity is mediated by ferroptosis with the proteinopathies either being epiphenomena or being upstream factors provoking ferroptosis is a hypothesis that we are aggressive studying currently. We hope that this approach may yield new therapeutic possibilities, and there are a few small clinical trials that lend some support to agents that have antiferroptosis properties. The NAC trial was mentioned above. A 24-weeks, multicenter, phase 2a, double-blinded randomized controlled clinical trial of selenite reported that the 30 mg/d dose was well tolerated in AD patients over 24 weeks and induced improved MRI signs (417) and less cognitive deterioration once adjusted for Se uptake into biofluids (418). Vitamin E and Se are components of a medical food that may slow cognitive deterioration in AD (419, 420, 421, 422).

## Conclusion

It has been just over 150 years since the elucidation of the Periodic Table, and more than 100 years since the identification of the first case of AD (423). Elements including zinc, copper, iron, and Se have been demonstrated by research to be closely involved in the pathogenesis of neurodegenerative diseases such as AD. The role of these elements in AD is not as simple as being factors that facilitate Aβ aggregation, as proposed in the early stages of this line of research. In contrast, their dysregulation may be a result of the disease and contribute to neuronal dysfunction or death in several aspects. Among the most important research questions related to the metal theory of disease is the investigation of the effects of pathogenic AD mutations on metal-related neurodegeneration. Presenilin, APP, and ApoE have each been associated with being influenced by or influencing the biological elements surveyed in this review. With the recognition of ferroptosis as a primordial form of regulated cell death and with the discovery of pathogenic and protective APP mutations modulating the retention of neuronal iron in a way that might concord with jeopardy to the neuron (304), the stage is now set for a systematic investigation of the impact of FAD mutations on ferroptosis. If FAD mutations promote ferroptosis, this could be an instance of antagonistic pleiotropy, where the risk of later life AD is offset by the protection against cancer and infection.

In this review, we have summarized our current evaluation of the most pertinent studies and ideas regarding the involvement of the essential elements in AD. Further investigations for the possibility of these elements to serve independently or synergistically as biomarkers of the disease or as therapeutic targets are warranted. The complex interplay between the metal supply systems and their protein targets has only been studied rudimentarily, *e.g.,* copper supply to Cp impacting iron efflux. An important take home message of this monograph is that pharmacological mechanisms that could modulate metalloneurobiological targets are multidimensional. Indeed, because the metals are central to essential biochemistry (*e.g.,* heme, respiratory chains, and antioxidants), high-affinity and nonselective chelators have some caveats as drug candidates. Compounds like PBT2 are low affinity metal-binding agents, and most antiferroptosis agents are not chelators at all. In one context, the supply of metals could be needed, and in another context, the withdrawal of metals is needed and neither can be readily achieved by dietary means because of the brain’s tenacious homeostatic systems for retaining essential elements. In yet another context, impaired turnover of metal ions, *e.g.,* zinc in the AD synapse is a critical pathophysiology and not remedied by high affinity chelation. Assimilating the information provided by elementomics and the interrogation of vital transition metals into our understanding of AD has evolved continually over the last 20 years (291, 424, 425, 426, 427). In this period, the dominant theories of AD have yet to provide a persuasively disease-modifying treatment. While the essential elements of AD have not garnered the attention that the proteinopathies of AD have enjoyed in this period, they remain essential and offer the promise of insights that may aid in the discovery of the first impactful disease-modifying drugs.

The ultimate formulation of AD will depend on what therapeutic targets are successful in clinical trials. At this point in time, the field is looking at targets outside of the dominating amyloid cascade hypothesis. The findings reviewed here show that the biological transition metals interact with the proteinopathy and the major gene products of AD in ways that make them plausible therapeutic targets. A multifactorial formulation of AD pathogenesis still seems most likely, but changes in these metals might yet be an upstream cause, a downstream consequence, or both. Most importantly, brain homeostasis of these metals changes with aging to feasibly explain why age is the major risk factor for AD. Whether one or all of these elements emerge as the major influence on AD pathogenesis will take research effort and the mobilization of some of the resources that can hopefully be liberated from the Sisyphean pursuit of amyloid as the culprit.

## Conflict of interest

A. I. B. is a shareholder in Alterity Therapeutics Ltd, Cogstate Ltd, and Mesoblast Ltd. He is a paid consultant for, and has a profit share interest in, Collaborative Medicinal Development Pty Ltd.
